# A dosimetry method for low dose rate brachytherapy by EGS5 combined with regression to reflect source strength shortage

**DOI:** 10.1093/jrr/rrt147

**Published:** 2014-01-20

**Authors:** Kenichi Tanaka, Kunihiko Tateoka, Osamu Asanuma, Ken-ichi Kamo, Kaori Sato, Hiromitsu Takeda, Masaru Takagi, Masato Hareyama, Jun Takada

**Affiliations:** 1 Department of Medicine, Graduate School of Sapporo Medical University, South 1, West 17, Chuo-ward, Sapporo, Hokkaido 060-8556, Japan; 2 Division of Radiology and Nuclear Medicine, Sapporo Medical University Hospital, South 1, West 17, Chuo-ward, Sapporo, Hokkaido 060-8556, Japan; 3 Hyogo Ion Beam Medical Center, 1-2-1, Kouto, Shingu, Tatsuno, Hyogo 679-5165, Japan; 4 Teishin-kai Radiation Therapy Institute, 1–6, North 44, East 8, Higashi-ward, Sapporo, Hokkaido 007-0844, Japan

**Keywords:** brachytherapy, dosimetry, source strength, glass rod dosimeter, EGS5, regression

## Abstract

The post-implantation dosimetry for brachytherapy using Monte Carlo calculation by EGS5 code combined with the source strength regression was investigated with respect to its validity. In this method, the source strength for the EGS5 calculation was adjusted with the regression, so that the calculation would reproduce the dose monitored with the glass rod dosimeters (GRDs) on a water phantom. The experiments were performed, simulating the case where one of two ^125^I sources of Oncoseed 6711 was lacking strength by 4–48%. As a result, the calculation without regression was in agreement with the GRD measurement within 26–62%. In this case, the shortage in strength of a source was neglected. By the regression, in order to reflect the strength shortage, the agreement was improved up to 17–24%. This agreement was also comparable with accuracy of the dose calculation for single source geometry reported previously. These results suggest the validity of the dosimetry method proposed in this study.

## INTRODUCTION

Brachytherapy has the advantage that the delivered dose can be controlled at intended positions directly by setting the position and strength of the source properly. Its dosimetry is performed with the American Association of Physicists in Medicine Task Group No. 43 Updated Protocol (AAPM-TG43U1) and its subsequent updates [1, 2]. In utilizing the TG43U1 formalism, accurate evaluation of the position and strength of the source is essential [3–5].

In interstitial low dose rate brachytherapy treatments, the sources generally move in the patient body after the implantation, due to the organ swelling, etc. After the movement, the source position is generally estimated using e.g. computed tomography. [6]. On the other hand, the source strength might have deviation from its nominal value. Thus, AAPM Task Group Reports 56 and 64 recommend that users measure the strength of 10% or more of the sources [7, 8]. In this protocol, 90% of the sources at most are utilized in treatments without measurement. This is due to the time-consuming task of measuring as many as tens to a hundred sources. Also, even if all the sources are successfully measured before the implantation, the possibility of human mistakes, such as accidental replacement of sources with those having incorrect strength, cannot be eliminated and its influence cannot be corrected in the treatment.

This study aims at developing a new post-implantation dosimetry option, where the source strength to be used for the dosimetry is adjusted by means of the regression, so that the calculated dose prediction will be reproduced by the experimental dose monitored after the implantation. This serves the purpose of an additional quality assurance (QA) activity to improve the dosimetry accuracy by considering the potential difference of the source strength from that in the treatment plan. This paper describes the validity of this methodology in a preliminary fashion.

## MATERIALS AND METHODS

The validity of the proposed methodology was tested by investigating its accuracy in estimating the dose distribution in a phantom having two internal sources. The difference of the strengths between the two sources was varied, in order to simulate the conditions in which one of the sources has a lower strength than that in the treatment plan. The regression was investigated as to its efficacy, i.e. how much the accuracy of the calculation was improved by the regression.

**Fig. 1.**
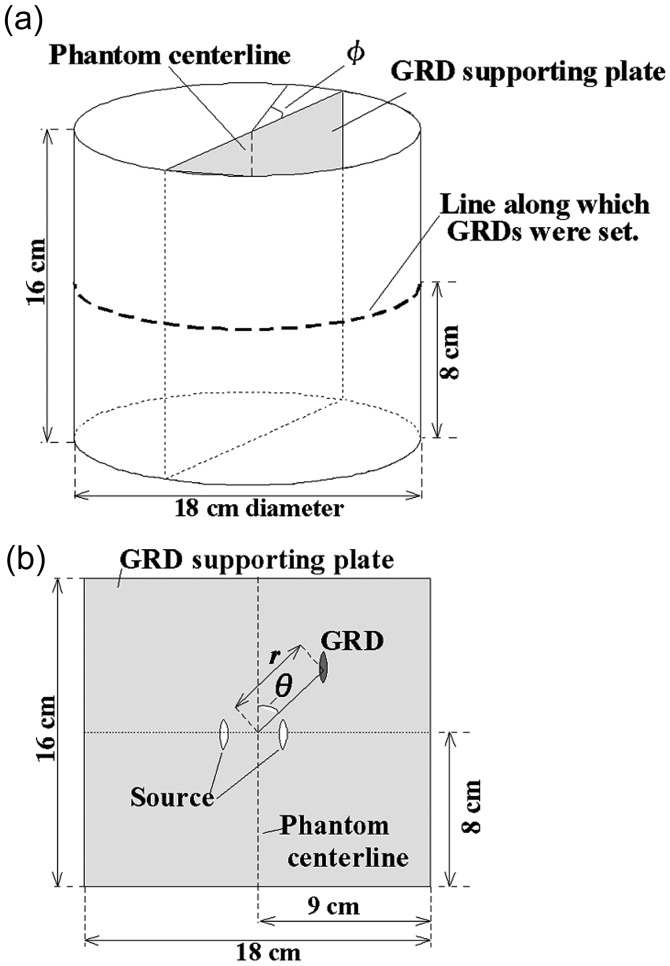
Geometry for measurement and calculation (**a**) overview, (**b**) cross sectional view.

The source utilized was the ^125^I seed (GE Healthcare Corporation, Oncoseed No. 6711). Its air kerma strength was measured with a well-type ionization chamber (Standard Imaging, Inc. HDR1000 Plus) to be 0.029–0.253 U. The dosimeters used were radiophotoluminescent glass rod dosimeters (GRDs) (Asahi Techno Glass Corporation, GD-302M), which were 1.5 mm in diameter and 12 mm in length. The phantom (Fig. 1) was a cylinder made of 2-mm thick polymethyl-methacrylate (PMMA) with a diameter of 18 cm and a length of 16 cm. The sources and GRDs were held in or on the phantom. The sources and GRDs in the phantom were located on the ‘GRD supporting plate’ made of 2-mm thick PMMA set along the phantom centerline, as shown in Fig. 1.

Two sources were set 1.5 cm away from the center of the phantom (*r* = 1.5 cm), at locations *θ* = 90° (#1) and 270° (#2). The GRDs for regression were set on the phantom surface along the dashed line in Fig. 1a with *ϕ* = 0, 45, 90, 135, 180, 225, 270 and 315°, and three GRDs were placed side by side at each specified angle of *ϕ*. As each GRD was set in a plastic casing of 2.8 mm in diameter, the neighboring GRDs have an angle difference of 0.9°. The average of the doses measured with three GRDs at *ϕ* − 0.9°, *ϕ*, *ϕ* + 0.9° was used as the dose at *ϕ*. The direction *ϕ* = 0° corresponds to *θ* = 90°. On the other hand, for the purpose of estimating the accuracy of the calculated doses, the GRDs were also placed in the phantom at the locations (*r* = 2, 4 and 6 cm, *θ* = 0 and 180°) and (*r* = 4, 6 and 8 cm, *θ* = 45, 135, 225 and 315°). In this case there was one GRD at each position.

The irradiation period was ∼ 24 h. The obtained result was the absorbed dose to glass. It was then converted to the absorbed dose to water, using the mass energy-absorption coefficient [9]. The details of the analysis and calibration are described previously [10].

The absorbed dose to water in/on the phantom was also calculated with the Monte Carlo (MC) code ‘EGS5′ [11], using the ^125^I source structure detailed by Kennedy *et al*. [12]. The obtained result was also the absorbed dose to glass, which was then converted to the absorbed dose to water, using the mass energy-absorption coefficient. Also, the calculated result was originally the dose per one photon emission. This was at first converted to the dose for the unit ‘actual activity’ using the photon emission rate of 1.5951 (photon/disintegration), then to the dose for the unit ‘apparent activity’ using the normalization factor of 1.8 obtained previously [10], and finally to the dose for the unit air kerma strength using the conversion factor of 1.27 (U/mCi) [13]. Here, the symbol U denotes the unit combination μGym^2^h^−1^, as defined in TG43U1 [1]. This calculation method was previously detailed in and validated in usage for the geometry lacking the equilibrium radiation scatter conditions in brachytherapy [10]. This method is also applicable to the interseed attenuation [14, 15] caused by other implanted sources, while TG43U1 is not applicable to these usages [10].

At first, the absorbed dose at the GRD positions was estimated using the aforementioned MC calculation for each of two sources having the unit air kerma strength. In this case, the calculation geometry included both of two source structures in order to consider interseed attenuation, however only one of them was assumed to emit radiations. Following this, in order for the MC calculation to reproduce the values measured with the GRDs placed on the phantom surface the source strength was determined with the regression. Finally, using the determined value as the source strength for the MC calculation, the dose distribution in the phantom was calculated. The result was compared with the values measured with the GRDs for estimating the accuracy; thus the efficacy of regression was investigated.

In regression, the dose measured with the GRDs, *D*_*exp*_(Gy), was fitted with a function corresponding to the linear combination of the calculated dose for source with the unit air kerma strength, *D*_*i cal*_(Gy/U), delivered by each of the sources at positions #1 and #2 given by:

(1) $$D_{exp} = a_{1}D_{1cal} + a_{2}D_{2cal}.$$

Here, *a*_*i*_ are the fitting parameters for each source. The parameters *a*_*i*_ also indicate the air kerma strengths (U) of the sources in calculation where the MC dose reproduces the measured dose. This function, which consists of linear combination of the calculated dose (Gy/U) and the source strength (U), is similar to that used in the treatment planning based on the TG43U1 formalism [1].

## RESULTS

**Fig. 2.**
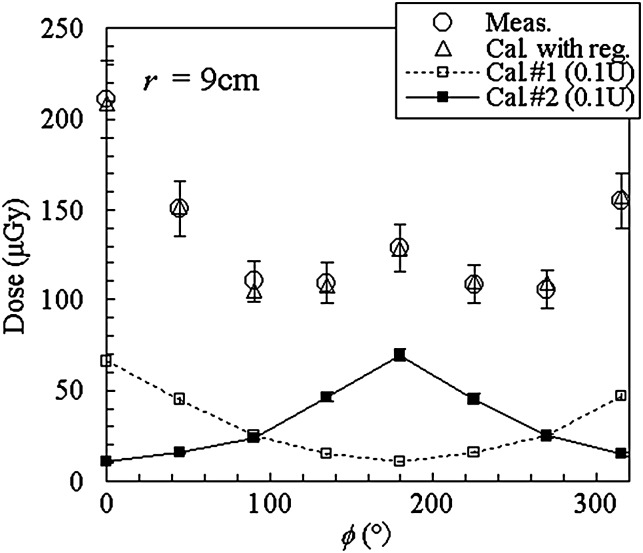
Dose distribution on phantom surface for Test 1. Calculated data for the sources at positions #1 and #2 are the values when one of the sources has the strength of 0.1 U and the other has 0 U.

In order to explain the data processing, the absorbed dose at GRD positions on the phantom surface is shown in Fig. 2 for ‘Test 1’. The calculated values for the sources at positions #1 and #2 are the absorbed dose normalized to the air kerma strength of 0.1 U to show the difference clearly. These dose distributions were fitted to the result of the GRD measurement to obtain the fitting parameters *a*_*i*_ in the equation (1). The dose distribution for estimated *a*_*i*_ is also shown in Fig. 2 as ‘Cal. with reg.’ The dose uncertainty was estimated similarly to the previous study [10], to be ∼10% for the measurement, ∼3–8% for the calculations with/without the regression. For the calculation, the uncertainty of the MC calculation was dominant while the uncertainty in estimating the fitting parameter was 1–3%. The uncertainties in this paper correspond to the combined standard uncertainty with the coverage factor of 1.

Table 1 summarizes the air kerma strengths estimated for four tests. The air kerma strengths exhibited as the ‘measurement’ are the values measured with the well-type chamber. The uncertainty was ∼3%, mainly by the documented uncertainty of 2.4% for the calibration of the chamber. The value in the parentheses is the ratio of the air kerma strength to that of the stronger source in each test. For example, in Test 1, the strength of the source at #2 corresponded to 52% of that of the source at #1. This is for the simulation of a treatment where the source at #1 has the same strength as that in the treatment plan, and the source at #2 lacks strength by 48%. Table 1 also lists the fitting parameters *a*_*i*_ in the equation (1) obtained by regression as the air kerma strengths for the ‘regression’. The exhibited uncertainty was that in estimating the fitting parameters of ∼1–3%. The source strength ratio in each test agreed between the chamber measurement and the regression approximately within the uncertainty. However, the regression is not for precise estimation of the source strength but that of the dose.

**Table 1.** Air kerma strength obtained by measurement and regression

	Source	Air kerma strength (U)			
	position	Measurement		Regression	
Test 1	#1 #2	0.253 ± 0.008 0.132 ± 0.004	(52 ± 2%)	0.291 ± 0.003 0.140 ± 0.003	(48 ± 1%)
Test 2	#1 #2	0.090 ± 0.003 0.062 ± 0.002	(69 ± 3%)	0.086 ± 0.002 0.064 ± 0.002	(75 ± 3%)
Test 3	#1 #2	0.046 ± 0.001 0.036 ± 0.001	(80 ± 3%)	0.047 ± 0.001 0.040 ± 0.001	(86 ± 3%)
Test 4	#1 #2	0.030 ± 0.001 0.029 ± 0.001	(96 ± 4%)	0.032 ± 0.001 0.031 ± 0.001	(96 ± 2%)

The air kerma strengths for the ‘measurement’ are the values measured with the well-type chamber, while those for the ‘regression’ are the values of the fitting parameters *a*_*i*_ in the equation (1) obtained by the regression. The value in the parenthesis is the ratio of air kerma strength to that of the stronger source in each test.

**Fig. 3.**
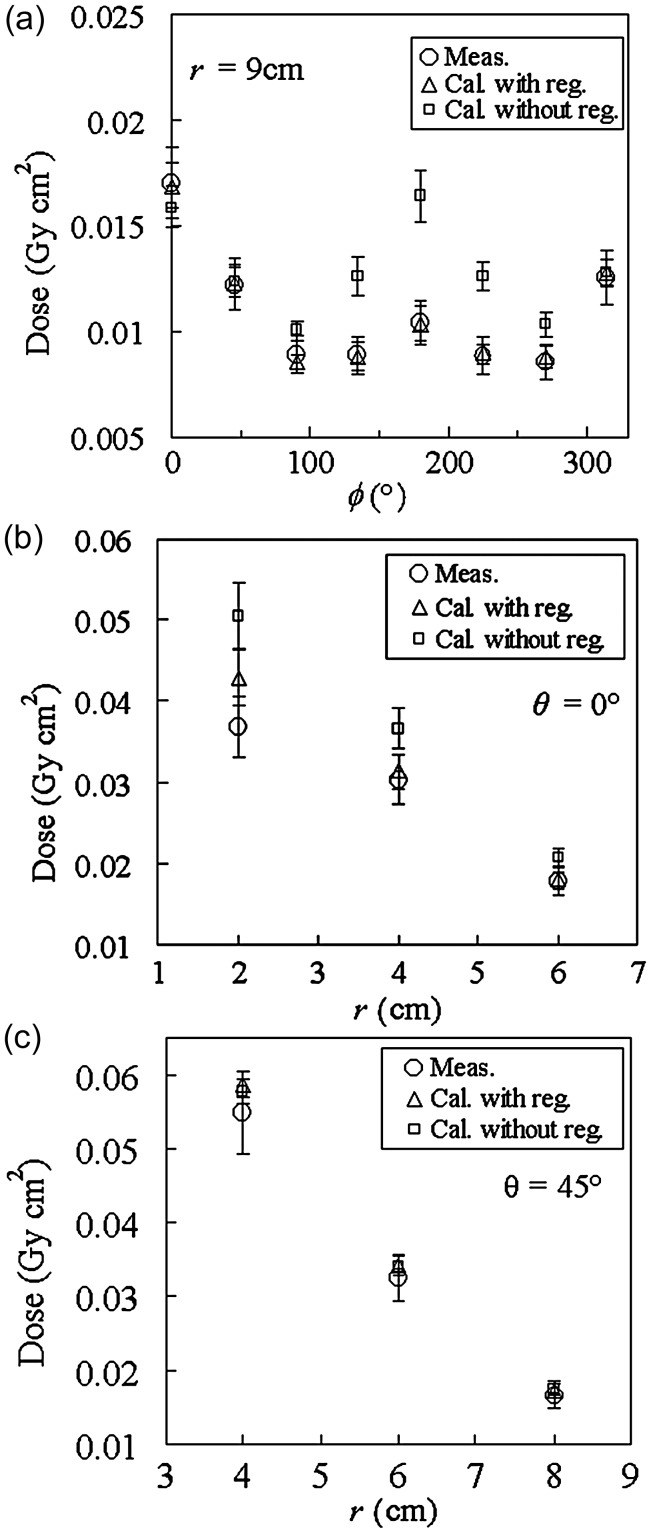
Comparison of dose in the phantom between GRD measurement and calculation with/without regression for Test 1: (**a**) on the phantom surface, (**b**) in the phantom at the directions of *θ* = 0°, and (**c**) at *θ* = 45°. The data are exhibited after being multiplied with the square of the phantom center to dosimeter distance (*r*) to show the data for large *r* clearly.

Figure 3 shows the dose distributions by the GRD measurement and calculation, with/without the regression for Test 1, (a) on the phantom surface, (b) in the phantom at the directions of *θ* = 0°, and (c) at *θ* = 45°. In order to show the data for large *r* clearly, the data are exhibited after being multiplied by the square of *r*. In the calculation without the regression, both sources were assumed to have the same strength as that in the treatment plan, i.e. 0.253 U. In this case, the calculation agreed with the measurement within 62%. On the other hand, in the calculation with the regression, the lack of the source at #2 in the strength was considered, and the agreement was within 17%.

Table 2 lists the agreement of the dose distributions in the phantom between the GRD measurement and the MC calculation combined with the source strength regression. In four tests with the source strength differences of 4–48%, the calculation without the regression agreed with the GRD measurement within 26–62%. By conducting the regression, the agreement was improved to 17–24%. Also, the resultant agreement is comparable with that for single source geometry, i.e. ∼25% [10]. These aspects support the validity of the presented calculation method and the usefulness of combining the regression.

**Table 2.** Agreement of dose distribution in the phantom between GRD measurement and calculation

	Range of dose ratio	(calulation/measurement)
	Without regression	With regression
Test 1	88–162%	87–117%
Test 2	87–152%	79–121%
Test 3	89–137%	87–124%
Test 4	74–109%	78–111%

## DISCUSSION

The proposed dosimetry method using the regression requires dose monitoring after implantation. This is to be carried out by keeping the GRDs on the body surface (in the form of clothing, etc.) for some time. By monitoring from outside the body, patients can be protected from an increase in load. In the present study, the phantom experiments were carried out over ∼24 h. In the treatment for prostate, for example, the number of the sources implanted are generally 80–100. In this case, the necessary irradiation time will be much shorter, such as 1 h. However, stable positioning of the GRDs is essential and the precision will influence the accuracy of the final estimated dose.

This study utilized the GRDs in the phantom. They are not intended to be used in actual treatments, but only for the accuracy test in this investigation. In practise, the patient dose is supposed to be estimated by the MC calculation, the source strength for which is adjusted by the regression so that the calculation will reproduce the dose monitored with the GRDs outside the body.

## CONCLUSION

In summary, the dosimetry determined by the MC calculation combined with source strength regression was investigated with respect to its validity. This was conducted with experiments simulating the situation where one of two sources lacked strength by 4–48%. As a result, performing the regression improved the agreement between the calculated and measured doses by up to 24%, which is comparable with that for single-source geometry. Combining the regression process of the source strength with dose estimation based on the MC calculation is useful in realizing an accurate dosimetry that reflects the potential difference of the source strength from that in the treatment plan. Since the calculation used was EGS5 code including the source structure in the geometry, the methodology proposed in this study is a potential option for a comprehensive dosimetry of brachytherapy including ISA and geometry lacking the equilibrium radiation scatter conditions.

## FUNDING

Part of the present study was supported by a Grant-in-Aid for Scientific Research from the Japan Society for the Promotion of Science under Grant #21791203, and the grant aid program by Sapporo Medical University, 2010.
